# Primary Melanoma Characteristics of Metastatic Disease: A Nationwide Cancer Registry Study

**DOI:** 10.3390/cancers13174431

**Published:** 2021-09-02

**Authors:** Catherine Zhou, Marieke Louwman, Marlies Wakkee, Astrid van der Veldt, Dirk Grünhagen, Cornelis Verhoef, Antien Mooyaart, Tamar Nijsten, Loes Hollestein

**Affiliations:** 1 Department of Dermatology, Erasmus MC Cancer Institute, Doctor Molewaterplein 40, 3015 GD Rotterdam, The Netherlands; c.zhou@erasmusmc.nl (C.Z.); m.wakkee@erasmusmc.nl (M.W.); t.nijsten@erasmusmc.nl (T.N.); 2 Department of Research and Development, Netherlands Comprehensive Cancer Organization, Godebaldkwartier 419, 3511 DT Utrecht, The Netherlands; m.louwman@iknl.nl; 3 Department of Medical Oncology and Radiology & Nuclear Medicine, Erasmus MC Cancer Institute, Doctor Molewaterplein 40, 3015 GD Rotterdam, The Netherlands; a.vanderveldt@erasmusmc.nl; 4 Department of Surgical Oncology, Erasmus MC Cancer Institute, Doctor Molewaterplein 40, 3015 GD Rotterdam, The Netherlands; d.grunhagen@erasmusmc.nl (D.G.); c.verhoef@erasmusmc.nl (C.V.); 5 Department of Pathology, Erasmus MC Cancer Institute, Doctor Molewaterplein 40, 3015 GD Rotterdam, The Netherlands; a.mooyaart@erasmusmc.nl

**Keywords:** melanoma, metastasis, disease progression

## Abstract

**Simple Summary:**

Melanoma of the skin is the most lethal form of skin cancer. Almost 40% of the patients who die of metastatic melanoma did not have metastases at first diagnosis. More knowledge about patient and tumour characteristics as well as patterns of disease progression is needed. We described the characteristics and disease patterns of early-stage melanomas that progress into metastatic disease. We observed that more than half of the patients with metastases were initially diagnosed with early-stage disease. Additionally, we found that melanomas in some specific body sites were likely to metastasize to certain organs. Our finding that a substantial proportion of patients with metastases were initially diagnosed with early-stage disease highlights the need to investigate who these high-risk patients are.

**Abstract:**

The characteristics and disease patterns of primary stage I and II cutaneous melanomas that progress to stage III or IV disease were investigated based on data from the Netherlands Cancer Registry (NCR). Data on stage III or IV melanomas at first diagnosis or during follow-up between 2017 and 2019 were retrieved. Patient and primary tumour characteristics were investigated in relation to time to disease progression and the number of organ sites with metastatic disease using regression models. In total, 2763 patients were included, of whom 1613 were diagnosed with stage IV disease. Among the patients with stage IV disease, 60% (*n* = 963) were initially diagnosed with stage I or II disease. The proportion of patients who received a sentinel lymph node biopsy increased after the introduction of adjuvant therapy in 2019 from 61% to 87%. Among all patients with stage III disease who were eligible for adjuvant systemic therapy (*n* = 453) after 2019, 37% were not treated with this therapy. Among patients with stage IV disease, lung metastases were most often detected as the first metastatic site and females presented with more metastatic sites than males. Most patient and primary tumour characteristics were not associated with the distant metastatic organ site, except melanoma localisation in the lower extremities and the head or neck. Our observation that most stage IV patients were initially diagnosed with early-stage disease highlights the need for more accurate risk prediction models.

## 1. Introduction

Cutaneous melanoma is the sixth most frequently diagnosed malignancy in Europe and the incidence rates are increasing in nearly all countries [1,2,3,4,5]. Around 90% of patients are initially diagnosed with stage I or II melanoma [5,6]. However, in 8% of the patients with stage I and 29% of the patients with stage II melanoma, the disease recurs within 5 years [7]. Because of the high proportion of stage I and II melanomas, the absolute numbers are substantial. Melanoma is one of the most aggressive types of skin cancer, and accounts for the majority of skin cancer deaths [1]. Worldwide, melanoma caused 57,074 deaths in 2020 [1]. Almost 40% of the patients who die of melanoma were initially diagnosed with stage I or II disease [8]. In most studies on disease progression of melanomas, the progression pattern is not described in relation to patient and tumour characteristics [7,9,10,11,12,13,14]. Data on the primary melanoma with follow-up to progression are scarce, because most cancer registries collect data on the primary diagnosis but not on disease progression during follow-up. Since mid-2017, the Netherlands Cancer Registry (NCR) routinely registers disease progression to stage III and IV in patients diagnosed with melanoma.

In recent years, the treatment of metastatic melanoma has undergone a revolution because of the introduction of adjuvant systemic therapy [15]. In January 2019, novel drugs for adjuvant systemic therapy for patients who were diagnosed with stage III and IV melanoma were approved by the Medicines Evaluation Board of the Dutch Society for Medical Oncology [16,17,18]. To identify patients at high risk for progression in an early stage and who may be eligible for systemic therapy, it is essential to gain more insight into patterns of disease progression based on primary tumour and patient characteristics. Therefore, we aimed to describe the primary tumour and patient characteristics of stage I and II cutaneous melanomas that progress to stage III or IV, using data from the NCR.

## 2. Materials and Methods

### 2.1. Setting and Patient Population

Data on patients with cutaneous melanoma who were diagnosed with stage III or IV disease at primary diagnosis or during follow-up between July 2017 and December 2019 were retrieved from the NCR. The selection was based on morphology codes M8720-8790 and topography code C44 of the third edition of the International Classification of Diseases for Oncology (ICD-O3). Melanomas of unknown primary site (C80.9) were excluded [19]. The NCR registers newly diagnosed malignancies upon automated notification by the nationwide network and registry of pathology since 1989. Since July 2017, data on patients with disease progression to stage III or IV melanoma are registered as well. Initially, patients with stage IV and inoperable stage IIIC/IIID disease were included in the database. From July 2018, the inclusion criteria were extended to all patients with stage IIIC/IIID disease. From January 2019, all patients with stage III and stage IV disease were included in the database. Eligibility for inclusion in the database based on stage III or IV disease was assessed according to the 8th edition of the staging system by the American Joint Committee on Cancer (AJCC) [20]. Trained data managers collected the data from pathology reports and digital patient records.

### 2.2. Patient and Tumour Characteristics

Data on gender, age, history of multiple primary melanomas, incidence date, topography, morphology, Breslow thickness, ulceration, clinical and pathological tumour-node-metastasis (TNM) stage, AJCC stage grouping valid at time of primary diagnosis, treatments, sentinel lymph node biopsy (SLNB), incidence dates of the first to the 10th recurrence or metastasis, type of recurrence and localisation of distant metastases were extracted. The incidence date of the primary melanoma was categorized into 5 categories: less than 2 years ago, 2–5 years ago, 5–10 years ago, 10–15 years ago and >15 years ago, as most melanomas recur within 5 years [7]. The topography of the primary cutaneous melanoma was categorized according to the ICD-O3 into face (C44.0, C44.1, C44.2 and C44.3), scalp and neck (C44.4), trunk (C44.5), upper extremities and shoulders (C44.6) and lower extremities and hips (C44.7). The Breslow thickness was categorized into ≤1.00 mm, 1.01–2.00 mm, 2.01–3.00 mm, 3.01–4.00 mm and ≥4.01 mm. The ulceration status was extracted from the pathological tumour (pT) stage according to the TNM and AJCC classifications that were valid at the time of diagnosis, if possible [21,22,23,24,25,26]. The clinical N stage (cN stage) was based on physical examination of the lymph nodes and may have been confirmed with imaging. The clinical M stage (cM stage) was based on physical examination, radiologic imaging, endoscopy or surgical exploration. Treatment of the initially diagnosed disease included surgical excision, lymph node dissection, radiotherapy, chemotherapy, targeted therapy and immune therapy. The latter also included locally injected viral therapy and dendritic cell therapy. In the NCR, SLNB outcome is classified as positive (tumour load > 2.0 mm), micrometastases present (0.21–2.0 mm), isolated tumour cells present (≤0.2 mm) or negative. The type of recurrence was classified as locoregional and/or distant. The localisation of distant metastases was registered according to the ICD-O3 and categorized into the major organs and tracts [19] (Supplementary Materials, Table S1). In case of stage IV disease, lymph node metastases only included distant lymph nodes, such as lymph nodes contralateral from the primary melanoma, and not locoregional lymph nodes. Metastases of the skin did not include in-transit and satellite metastases.

### 2.3. Statistical Analysis

Descriptive patient and tumour characteristics were stratified for stage III or IV disease at primary diagnosis or during follow-up. The NCR collects data on SLNBs since January 2014. Therefore, patients diagnosed before 2014 were excluded for all SNLB analyses. The trends in SLNBs were described by stratification of the time period before and after adjuvant systemic therapy became available for stage IIIA patients with positive lymph nodes of >1.0 mm and stage IIIC-D patients in January 2019 in the Netherlands. The proportion of patients who had an SLNB was described for all eligible patients (i.e., stage pT1b without clinical suspicion of metastases (cN0 and cM0)) [27].

Adjuvant systemic therapy was described for all eligible patients (i.e., stage IIIA patients with positive lymph nodes of >1.0 mm and stage IIIC-D patients). Chi-square tests were used to compare characteristics between treated and untreated patients.

For all analyses regarding disease progression, we included patients who were diagnosed with a single stage I-III primary melanoma after July 2017, to ensure that the first stage IV presentation was analysed. Model assumptions of all regression analyses described below were checked and met. A linear regression model was used to investigate the association between the time to first distant metastasis (dependent variable) and the number of metastatic organ sites (independent variable). A linear mixed-effects model was used to investigate the association between time to progression (dependent variable) and localisation of distant metastases (independent variable), because a patient may develop multiple recurrences. The first distant recurrence of each localisation was included in the analyses. The model included a random effect for each patient. To investigate the association between the number of metastatic organ sites in the first stage IV presentation (dependent variable) and patient and tumour characteristics (independent variables), a multivariable Poisson regression model was used. For all covariates, the largest category was the reference.

All patients with stage IV disease at initial diagnosis or during follow-up were included to investigate the association between the localisation of distant metastases (dependent variable) and patient and tumour characteristics (independent variables). Univariable and multivariable multinomial logistic regression analyses were performed to calculate odds ratios for each organ site affected by metastases. All first distant metastases of each localisation were included. Organ sites were compared with the organ site that was most often affected by metastases. Missing values were imputed twenty times using multiple imputation via chained equations and predictive mean matching. The imputation model included all patient and tumour characteristics as well as the type of affected organ. In this analysis, we controlled the false discovery rate by calculating corrected levels of significance using the method described by Benjamini and Hochberg [28].

All analyses were conducted using IBM^®^ SPSS^®^ Statistics 25.0. *p*-values were considered significant if *p* < 0.05 (two-sided).

## 3. Results

### 3.1. Patient and Tumour Characteristics

In total, 2763 patients with a stage III or IV melanoma were included in the NCR between July 2017 and December 2019 (Table 1). Of all patients, 1612 (58%) patients were male and 1151 (42%) were female. The median age at diagnosis of the primary tumour was 63 years (interquartile range: 52–73 years). The majority of the primary melanomas were diagnosed less than 5 years before progression to stage III or IV. Almost half of the patients diagnosed with stage III or IV melanoma (*n* = 1346 (49%)), were initially diagnosed with stage I or II melanoma. Among patients with stage IV disease, this percentage was 60% (*n* = 963). Of patients diagnosed with stage III melanoma, 56% (*n* = 648) were diagnosed with this disease stage at first presentation. Of patients diagnosed with stage IV melanoma, this figure was 14% (*n* = 223) (Table 1).

### 3.2. Trends in Sentinel Lymph Node Biopsy

Between January 2014 and December 2019, where data on SLNBs were collected, 2130 patients were diagnosed with a primary melanoma. From the 1462 patients who were diagnosed before the introduction of adjuvant therapy for stage III melanoma (January 2014 until December 2018), 61% of the 1156 patients eligible for an SLNB had an SLNB (Table 2). The proportion of eligible patients who received an SLNB increased to 87% (*n* = 434) after the introduction of adjuvant therapy (January 2019 until December 2019). Whilst the proportion of patients who received an SLNB increased with T stage to stage T3a, the proportion of SLNBs decreased among thicker melanomas (Table 2). Furthermore, an increase in the proportion of patients with a positive sentinel node was observed, from 29% to 38%, as well as in the proportion of patients with micrometastases (15% to 39%) and isolated tumour cells (4% to 12%) in the sentinel node (Table S2).

### 3.3. Adjuvant Systemic Therapy

In total, 62% (*n* = 281) of all eligible stage III patients (*n* = 453) received adjuvant systemic therapy after January 2019 (Table 3). The majority of these eligible patients that were treated with adjuvant immune therapy were diagnosed with stage IIIC disease (56%, *n* = 158). Almost half of the patients who were not treated with adjuvant immune therapy were 71 years or older (47%, *n* = 82 and *p* < 0.001). No major differences were found in treatment with immune therapy among other patient and tumour characteristics (Table 3).

### 3.4. Time to Progression

In total, 234 (58%) patients with a stage I, II or III single primary melanoma diagnosed after July 2017 developed distant metastases during follow-up (Table 4). Seventeen percent of these patients progressed from stage I or II to stage IV directly. Sixteen percent of these patients had metastases in the distant lymph nodes only, without haematogenous metastases. Most of the patients (40%, *n* = 102) had metastases in one organ site, and less than 20 patients had metastases in six or more organ sites at first stage IV presentation. There was no statistically significant difference in the mean time to progression of patients with metastases in multiple organ sites compared to a single site of distant metastases (β per extra organ site = −0.1, 95% CI: −0.6 to 0.3, *p* = 0.66) (Table 4).

To study time to metastasis for each organ site, we included all first metastases of each organ site (*n* = 974) of the aforementioned 234 patients. Of all organ sites, the first distant metastases were found most often in the lungs (20%, *n* = 195), followed by distant lymph nodes (15%, *n* = 145), the liver (14%, *n* = 135) and the brain (14%, *n* = 131) (Table 4). Metastases were detected later in time in the brain, connective tissue and gastrointestinal tract than in the lungs (+1.3 months (95% CI: 0.8 to 1.9 months), +0.8 months (95% CI: 0.2 to 1.5 months) and +2.3 months (95% CI: 1.1 to 3.5 months), respectively). There was no significant difference in mean time to metastases to other organ sites (Table 4). The mean time to distant metastases did not significantly differ among patient and tumour characteristics (Table S3).

### 3.5. Number of Metastatic Organ Sites

On average, patients presented with 2.4 metastatic organ sites (SD: 1.6) at their first stage IV diagnosis (Table 5). Females presented with more affected sites than males (ratio: 1.15, 95% CI: 1.01 to 1.30, *p* = 0.03). Furthermore, patients who were 51–60 years old at primary diagnosis presented with more metastatic sites than patients in other age categories (ratio: 1.34, 95% CI: 1.12 to 1.60, *p* < 0.001). Other patient and tumour characteristics were not significantly associated with the mean number of affected organ sites (Table 5).

### 3.6. Localisation of Metastatic Organ Sites

In total, 4774 first distant metastases for each localisation were included. Nineteen percent (*n* = 914) were lung metastases (Table S4).

After correcting for multiple comparisons, we observed that distant metastases of the lymph nodes, connective tissue and the skin originated more frequently from the lower extremities compared to the body site distribution of primary melanomas that metastasized to the lungs (adjusted OR: 2.0 (95% CI: 1.5–2.6), adjusted OR: 1.6 (95% CI: 1.1–2.2) and adjusted OR: 1.9 (95% CI: 1.9–4.7), respectively) (Table 6, Table S5). Metastases of the heart or pleura originated more frequently from the head or neck area compared to the body site distribution of primary melanomas that metastasized to the lungs (OR: 2.1, 95% CI: 1.3–3.4). Other results did not remain statistically significant after the correction of the significance level for multiple comparisons (Table 6, Table S5).

## 4. Discussion

In our nationwide cohort of patients with metastatic melanoma, we found that half of these patients did not have any metastasis at the date of primary diagnosis. Of all patients who developed distant metastases, this percentage was even higher (60%). We did not observe that melanomas with metastases to multiple distant sites at once metastasize earlier compared to melanomas that metastasize to a single site. Metastases in the lungs occurred earlier in time than metastases in the brain, connective tissue or gastrointestinal tract. Additionally, female patients presented metastases in more distant organ sites than male patients at first diagnosis of stage IV disease. Lastly, primary melanomas in the lower extremities were more likely to metastasize to the distant lymph nodes, connective tissue and skin than to the lungs, compared to primary melanomas in the trunk. Primary melanomas in the head or neck area were more likely to metastasize to cardiac tissue or the pleura.

In other studies, it was observed that 31% to 64% of melanomas with distant metastases were initially diagnosed at an early stage [8,29,30]. Our estimation of 60% is in the high range. We also observed that before the introduction of adjuvant therapy, almost 40% of all patients who were eligible for an SLNB did not undergo this procedure. Therefore, some lymph node metastases may have been missed and melanomas may have been incorrectly staged as stage I or II. Another possible explanation is that the guidelines for the diagnostic work-up at primary diagnosis differ among countries. In contrast to the Dutch guidelines, the American Academy of Dermatology recommends consultations with oncologists for discussion of surveillance imaging for stage IIB and IIC melanomas [27,31]. This may result in a higher detection rate of metastatic disease at first diagnosis and thus a lower proportion of melanomas that were initially early-stage in the aforementioned American studies [8,29]. In a recent study that compared incidence and survival of melanoma between two neighbouring countries, Belgium and the Netherlands, the authors observed a higher incidence rate of stage IV disease at first diagnosis in Belgium than in the Netherlands [32]. The Belgian guidelines recommend ultrasonography of the draining lymph nodes and the abdomen as well as chest radiography from stage T1b onwards [33], whereas the Dutch guidelines do not recommend routine supplementary imaging for stage I-IIIA patients [27].

Due to the availability of adjuvant therapy for stage III disease, SLNBs became increasingly important. This led to an increase in the proportion of eligible patients that had an SLNB from 61% before 2019 to 87% after January 2019. As expected, the number of complete lymph node dissections (CLNDs) decreased, due to the results of a MSLT-II trial in 2017 [34]. Controversially, a lower proportion of patients who received an SLNB was observed in higher pT stages (71% in pT4b vs. 95% in pT2b). A possible explanation is that additional imaging is performed more frequently in these thicker tumours [27]. Additionally, an increased proportion of positive SNs was observed (29% to 38%), along with an increased proportion of isolated tumour cells (4% to 12%) and micrometastases (15% to 39%). Newly developed histopathologic work-up protocols that include transhilar bivalving and microsections from the central hilar planes may have resulted in a higher positive detection rate for these outcomes [35,36,37].

We observed that 38% of all eligible stage III patients were not treated with adjuvant immune therapy and that patients of 71 years were less likely to receive adjuvant immune therapy. Factors that are important in shared decision-making of adjuvant therapy for melanoma are comorbidities, quality of life, side effects and the patient’s wishes [38].

In accordance with other studies, we observed that the most common site of distant metastases is the lungs and that metastases in the lungs occur relatively early in time [39,40,41,42]. In accordance with the aforementioned studies, we also observed that metastases in the distant lymph nodes and liver occurred frequently. We observed that metastases in the brain, bone and gastrointestinal tract occurred later in time. A possible explanation is that lung metastases are diagnosed earlier and more frequently, because chest X-rays are more frequently performed than other types of imaging, such as cerebral MRIs or colonoscopies, to detect brain metastases and gastrointestinal metastases, respectively. A difference in the tumour microenvironment between the organ sites may play a role as well. Lung metastases are common for many malignancies, perhaps because blood and lymphatic fluid returning from the periphery is first pumped through the pulmonary vasculature [41]. More specifically for melanoma, it has been found in murine models that organ-specific extracellular proteins in the lungs and chemokine receptors, such as CXCR4, in the primary melanoma may mediate the preferential metastases in the lungs [43,44]. Brain metastases are also frequent, but often occur when metastases at other sites are already present [45]. The later occurrence of metastases in the brain may be caused by the non-fenestrated endothelial lining in the brain that functions as a blood-brain barrier [46,47]. Additionally, the different microenvironmental conditions in the brain make it so that melanoma cells need to modify in order to survive [47]. Our finding that metastases in the brain, bone and the gastrointestinal tract occur relatively late in melanoma progression is also accordant with other studies [41,45]. Late metastases in certain organs may indicate that more mutations need to be accumulated in order to adapt to the new tumour microenvironment.

It has been widely studied that women have “somewhat less malignant” melanomas than men [48,49]. Gender has been confirmed as an independent predictive factor in stage III and IV melanoma after adjusting for other known factors, such as lifestyle, age and Breslow thickness. This has suggested that biological differences between men and women may play a role [50]. However, the underlying mechanisms of this female advantage remain unclear. Our finding that women present with more organ sites affected by metastases at their first stage IV diagnosis than men may be contradictory, especially because it has been shown that patients with single-organ metastases have significant survival advantages over patients with multiple metastases [30]. Nevertheless, the biological advantage that women have seems to persist even after adjustment for the number of affected organ sites [50].

Results from a small study among 92 patients suggested that cutaneous melanoma metastasizes to arbitrary organs without any association with patient and tumour characteristics [51]. In our large study, we observed a few associations between the metastatic site and patient as well as primary tumour characteristics. Lymph node, connective tissue and skin metastases were twice as likely to originate from primary melanomas in the lower extremities in comparison to the distribution of body site among lung metastases. Metastases of cardiac or pleural tissue originated more often from head/neck melanomas than lung metastases in comparison to the distribution of body site among lung metastases. External validation of our results is recommended to confirm if this is a general pattern. Targeted imaging may be considered if a general pattern can be validated.

A key strength of our study is the routine collection of follow-up data on all melanoma patients who have disease progression to stage III and IV, irrespective of whether the patients received novel therapeutic agents. Another strength is that the inclusion of patients is not limited to those patients with histologically confirmed metastases, but that patients without histologically confirmed metastases were included via notification by the Dutch Hospital Database.

Limitations of our study include the fact that follow-up records of one of all fourteen melanoma centers were absent in the NCR, that the NCR did not register follow-up records before mid-2017 and that the inclusion criteria changed. Therefore, we restricted the patient selection for some analyses to assure complete follow-up, but this resulted in limited follow-up time. Future studies should investigate disease progression patterns among patients who received adjuvant immunotherapy, when follow-up time is sufficiently long.

## 5. Conclusions

In conclusion, we described the patient, primary tumour and treatment characteristics of metastatic melanoma. We observed that SLNB was applied more frequently since the introduction of adjuvant therapy and that older patients were less likely to receive adjuvant therapy. The patterns of melanoma progression that we observed should be externally validated and may give rise to new studies with the aim of unravelling the biological mechanisms of differences in time to progression to different organ sites and their association with primary tumour characteristics.

Our most important observation is that more than half of the patients with distant metastases were initially diagnosed with stage I or II melanoma. Although most patients with stage I or II disease have a favourable prognosis, our results show that the absolute number of patients who develop metastases within this group is substantial. The shift towards adjuvant systemic therapy for more early-stage patients makes the priority to develop accurate strategies to identify stage I and II patients who are at high risk for disease progression even higher.

## Figures and Tables

**Table 1:** Patient and tumour characteristics of the primary melanoma of patients with stage III or IV disease.

	All Patients with Stage III or IV Disease	Patients with Stage III Disease	Patients with Stage IV Disease
Characteristics of the Primary Melanoma	(*n* = 2763)	(*n* = 1150)	(*n* = 1613)
**Gender, No. (%)**			
Male	1612 (58)	617 (54)	995 (62)
Female	1151 (42)	533 (46)	618 (38)
**Age at Diagnosis, Median (IQR ^1^) Years**	63 (52–73)	65 (53–74)	63 (51–72)
**Time since Primary Melanoma Diagnosis, No. (%)**			
Stage III or IV at first presentation	871 (32)	648 (56)	223 (14)
≤2 years ago	932 (34)	272 (24)	660 (41)
2–5 years ago	481 (17)	112 (10)	369 (23)
5–10 years ago	279 (10)	65 (6)	214 (13)
10–15 years ago	109 (4)	28 (2)	81 (5)
≥15 years ago	91 (3)	25 (2)	66 (4)
**Site of Primary Melanoma, No. (%)**			
Face	186 (7)	74 (6)	112 (7)
Scalp and neck	233 (8)	79 (7)	154 (10)
Trunk	1158 (42)	438 (38)	720 (45)
Upper extremities and shoulders	430 (16)	179 (16)	251 (16)
Lower extremities and hips	749 (27)	380 (33)	369 (23)
**Histopathological Subtype, No. (%)**			
Superficial spreading	1559 (56)	670 (58)	889 (55)
Nodular melanoma	725 (26)	306 (27)	419 (26)
Malignant melanoma unspecified	311 (11)	106 (9)	205 (13)
Other	98 (4)	28 (2)	70 (4)
Acral lentiginous melanoma	70 (3)	40 (3)	30 (2)
**Breslow Thickness, No. (%)**			
≤1.00 mm	354 (13)	115 (10)	239 (15)
1.01–2.00 mm	692 (25)	283 (25)	409 (25)
2.01–4.00 mm	814 (29)	372 (32)	442 (27)
≥4.01 mm	726 (26)	331 (29)	395 (25)
Unknown	177 (6)	49 (4)	128 (8)
**Ulceration Status, No. (%)**			
Ulcerated	960 (35)	418 (36)	542 (34)
Not ulcerated	1436 (52)	620 (54)	816 (51)
Unknown	367 (13)	112 (10)	255 (16)
**Stage (AJCC ^2^ Edition Valid at Time of Diagnosis), No. (%)**			
IA	189 (7)	50 (4)	139 (9)
IB	424 (15)	101 (9)	323 (20)
IIA	302 (11)	88 (8)	214 (13)
IIB	265 (10)	88 (8)	177 (11)
IIC	166 (6)	56 (5)	110 (7)
IIIA	253 (9)	162 (14)	91 (6)
IIIB	301 (11)	177 (15)	124 (8)
IIIC	541 (20)	370 (32)	171 (11)
IIID	37 (1)	19 (2)	18 (1)
IV	153 (6)	0 (0)	153 (9)
Unknown	132 (5)	39 (3)	93 (6)
**History of Multiple Primary Melanomas, No. (%)**	211 (8)	82 (7)	129 (8)
**Treatments Initiated for Primary Melanoma, No. (%)**			
Surgical excision	2699 (98)	1136 (99)	1563 (97)
Lymph node dissection	353 (13)	122 (11)	231 (14)
Adjuvant systemic therapy	514 (19)	338 (29)	176 (11)
Chemotherapy	11 (0)	4 (0)	7 (0)
Radiotherapy	80 (3)	15 (1)	65 (4)

^1^, interquartile range; ^2^, American Joint Committee on Cancer.

**Table 2:** Sentinel lymph node biopsies (SLNBs) performed before (2014–2018) and after (2019) the implementation of adjuvant systemic therapy, and the proportion of patients with positive macro-metastasized lymph nodes.

	Before the Implementation of Adjuvant Systemic Therapy			After the Implementation of Adjuvant Systemic Therapy		
	Total, No.	SLNBs Performed, No. (% of Total)	SLNB-Positive, No. (% of SLNBs Performed)	Total, No.	SLNBs Performed, No. (% of Total)	SLNB-Positive, No. (% of SLNBs Performed)
All patients regardless of eligibility for an SLNB	1462	754 (52)	231 (31)	668	464 (70)	185 (40)
No clinical suspicion for metastases	1258	719 (57)	209 (29)	518	442 (85)	168 (38)
No clinical suspicion for metastases stage ≥ pT1b	1156	705 (61)	204 (29)	498	434 (87)	163 (38)
**Pathological T Stage of Patients without Clinical Suspicion for Metastases**						
pT1a	70	3 (4)	0 (0)	7	2 (29)	2 (100)
pT1b	69	31 (45)	2 (7)	33	31 (93)	7 (23)
pT2a	222	131 (59)	25 (19)	122	114 (93)	28 (25)
pT2b	69	46 (67)	10 (22)	21	20 (95)	6 (30)
pT3a	213	142 (67)	46 (32)	96	89 (93)	32 (36)
pT3b	205	145 (71)	49 (34)	80	70 (88)	35 (50)
pT4a	144	82 (57)	31 (38)	47	40 (85)	19 (48)
pT4b	234	128 (55)	43 (34)	99	70 (71)	36 (51)
Unknown	32	11 (34)	3 (27)	13	6 (46)	3 (50)
Total complete lymph node dissections performed, no.	197			156		
Complete lymph node dissection performed, no. (% of all SLNB-positive patients)	91 (39)			50 (27)		

**Table 3:** Adjuvant systemic therapy received by patients diagnosed with a stage III primary melanoma who were eligible for adjuvant systemic therapy after January 2019.

	Eligible for Immune Therapy	Treated with Immune Therapy	Untreated with Immune Therapy	
	(*n* = 453)	(*n* = 281)	(*n* = 172)	*p*-Value
**Stage at Primary Diagnosis, No (%)**				
IIIA and positive sentinel node >1 mm	44 (10)	30 (11)	14 (8)	0.04
IIIB	137 (30)	79 (28)	58 (34)	
IIIC	257 (57)	158 (56)	99 (58)	
IIID	15 (3)	14 (5)	1 (4)	
**Gender, No. (%)**				
Male	273 (60)	173 (62)	100 (58)	0.47
Female	180 (40)	108 (38)	72 (42)	
**Age at Diagnosis, No. (%)**				
<40	34 (8)	28 (10)	6 (3)	<0.001
41–50	49 (11)	34 (12)	15 (9)	
51–60	106 (23)	71 (25)	35 (20)	
61–70	113 (25)	79 (28)	34 (20)	
≥71	151 (33)	69 (25)	82 (47)	
**Breslow Thickness, No. (%)**				
<1.00 mm	24 (5)	14 (5)	10 (6)	0.69
1.01–2.00 mm	74 (16)	51 (18)	23 (13)	
2.01–4.00 mm	170 (40)	106 (38)	73 (42)	
>4.01 mm	163 (36)	102 (36)	61 (40)	
Unknown	13 (3)	8 (3)	5 (3)	
**Ulceration Status, No. (%)**				
Ulcerated	205 (45)	132 (47)	86 (50)	0.54
Not ulcerated	219 (48)	133 (47)	73 (42)	
Unknown	29 (6)	16 (6)	13 (8)	

**Table 4:** The mean time interval from diagnosis of primary melanoma to progression to first stage IV presentation related to the number of sites and the localisation of metastases of patients diagnosed with a single stage I-III primary melanoma between July 2017 and December 2019.

	No.	Mean Time Interval, Months (SD ^1^)	Difference in Months	95% CI ^2^	*p*-Value
No. of stage I-III patients with progression to stage IV	234				
Total no. of sites of distant metastases	974				
**Number of Metastatic Organ Sites at First Stage IV Presentation**					
1 site	92	9.9 (5.6)	−0.1 per site	−0.6 to 0.4	0.66
2 sites	46	11.1 (7.2)			
3 sites	33	11.4 (5.9)			
4 sites	31	10.5 (5.2)			
5 sites	14	12.9 (6.1)			
≥6 sites	18	8.8 (8.0)			
**Time to Progression for each Site of Distant Metastases**					
Lung	195	10.0 (6.1)	ref		
Lymph node, distant	145	10.7 (6.3)	0.3	−0.2 to 0.8	0.20
Liver	135	9.8 (6.0)	−0.2	−0.3 to 0.8	0.77
Brain	131	11.4 (6.2)	**1.2**	**0.6 to 1.7**	**<0.001**
Bone	110	10.1 (6.1)	0.0	−0.6 to 0.5	0.99
Connective tissue	67	11.1 (7.1)	**0.7**	**0.0 to 1.3**	**0.05**
Other	45	9.6 (6.8)	−0.1	−0.9 to 0.7	0.79
Spleen	34	7.7 (3.7)	−0.5	−1.4 to 0.4	0.27
Adrenal gland	25	8.6 (5.8)	0.4	−0.6 to 1.3	0.45
Heart, pleura	24	10.7 (5.6)	−0.1	−1.2 to 0.9	0.96
Subcutis	24	6.5 (4.8)	−0.7	−1.8 to 0.3	0.18
Peritoneum	20	10.0 (6.3)	0.4	−0.7 to 1.4	0.49
Digestive system	19	12.9 (6.8)	**1.8**	**0.7 to 3.0**	**0.002**

^1^, standard deviation; ^2^, confidence interval.

**Table 5:** The mean number of metastatic organ sites with distant metastases at first presentation of stage IV disease in relation to patient and tumour characteristics in patients diagnosed with a single stage I–III primary melanoma between July 2017 and December 2019 in a multivariate model.

	No.	Mean Number of Sites (SD ^1^)	Ratio of No. of Metastatic Sites	95% CI ^2^	*p*-Value
All stage I-III patients with progression to stage IV	234	2.4 (1.6)			
**Gender**					
Male	149	2.3 (1.6)	1.0 (ref)		
Female	85	2.9 (2.0)	**1.15**	**1.01 to 1.30**	**0.03**
**Age at Diagnosis**					
≤40	10	2.8 (2.7)	1.09	0.81 to 1.45	0.58
41–50	25	2.4 (1.9)	1.00	0.82 to 1.22	0.10
51–60	30	3.3 (1.9)	**1.34**	**1.12 to 1.60**	**<0.001**
61–70	66	2.3 (1.6)	1.03	0.89 to 1.19	0.69
≥71	103	2.5 (1.6)	1.0 (ref)		
**Site of Primary Melanoma**					
Face	24	2.5 (1.7)	1.05	0.85 to 1.29	0.66
Scalp and neck	31	2.6 (2.1)	0.97	0.81 to 1.17	0.78
Trunk	104	2.6 (1.7)	1.0 (ref)		
Upper extremities and shoulders	25	2.5 (1.7)	1.01	0.84 to 1.23	0.90
Lower extremities and hips	49	2.4 (1.7)	−0.86	0.73 to 1.02	0.86
**Histopathological Subtype**					
Superficial spreading	127	2.7 (1.7)	1.0 (ref)		
Nodular melanoma	68	2.6 (1.8)	0.97	0.84 to 1.12	0.70
Acral lentiginous melanoma	4	1.3 (0.5)	0.78	0.49 to 1.25	0.30
Other	13	2.3 (1.7)	0.80	0.59 to 1.10	0.16
Malignant melanoma unspecified	22	2.1 (1.9)	0.90	0.74 to 1.10	0.31
**Breslow Thickness**					
≤1.00 mm	16	2.6 (1.8)	1.02	0.79 to 1.31	0.80
1.01–2.00 mm	46	2.5 (1.6)	0.98	0.81 to 1.19	0.81
2.01–4.00 mm	67	2.6 (1.5)	0.96	0.79 to 1.31	0.89
≥4.01 mm	91	2.6 (1.8)	1.0 (ref)		
Unknown	14	2.5 (2.2)	**1.30**	**1.06 to 1.60**	**0.03**

^1^, standard deviation; ^2^, confidence interval.

**Table 6:** The localisation of distant metastases in relation to patient and tumour characteristics in a multivariable model with the lungs as the reference metastatic site.

	Lymph Node	Liver	Brain	Bone	Connective Tissue	Other	Spleen	Adrenal Gland	Heart or Pleura	Skin	Peritoneum	Digestive System
	OR (95% CI)	OR (95% CI)	OR (95% CI)	OR (95% CI)	OR (95% CI)	OR (95% CI)	OR (95% CI)	OR (95% CI)	OR (95% CI)	OR (95% CI)	OR (95% CI)	OR (95% CI)
**Gender**												
Male	1.0 (ref)	1.0 (ref)	1.0 (ref)	1.0 (ref)	1.0 (ref)	1.0 (ref)	1.0 (ref)	1.0 (ref)	1.0 (ref)	1.0 (ref)	1.0 (ref)	1.0 (ref)
Female	0.8 (0.7–1.0)	0.9 (0.7–1.1)	1.0 (0.8–1.3)	0.9 (0.6–1.1)	1.3 (1.0–1.7)	1.1 (0.8–1.5)	**0.6 (0.4–1.0)**	0.8 (0.5–1.2)	1.2 (0.8–1.8)	0.9 (0.6–1.3)	1.0 (0.7–1.4)	1.0 (0.7–1.4)
**Age at Diagnosis**	1.0 (1.0–1.0)	1.0 (1.0–1.0)	**1.0 (1.0–1.0)**	1.0 (1.0–1.0)	1.0 (1.0–1.0)	1.0 (1.0–1.0)	1.0 (1.0–1.0)	1.0 (1.0–1.0)	1.0 (1.0–1.0)	1.0 (1.0–1.0)	1.0 (1.0–1.0)	1.0 (1.0–1.0)
**Site of Primary Melanoma**												
Head/neck	0.8 (0.6–1.1)	1.1 (0.8–1.5)	1.0 (0.8–1.4)	1.1 (0.8–1.5)	0.9 (0.6–1.4)	0.9 (0.6–1.4)	1.1 (0.6–1.8)	0.7 (0.5–1.2)	2.1 (1.3–3.4) *	0.9 (0.5–1.7)	0.6 (0.3–1.0)	0.6 (0.4–1.0)
Trunk	1.0 (ref)	1.0 (ref)	1.0 (ref)	1.0 (ref)	1.0 (ref)	1.0 (ref)	1.0 (ref)	1.0 (ref)	1.0 (ref)	1.0 (ref)	1.0 (ref)	1.0 (ref)
Upper extremities	1.1 (0.8–1.4)	1.0 (0.7–1.4)	1.0 (0.7–1.3)	1.2 (0.9–1.7)	1.3 (0.8–1.8)	0.9 (0.6–1.4)	1.2 (0.7–2.0)	1.2 (0.8–1.9)	1.1 (0.6–2.0)	1.5 (0.9–2.7)	1.0 (0.6–1.6)	0.7 (0.4–1.2)
Lower extremities	2.0 (1.5–2.6) *	1.2 (0.9–1.6)	1.0 (0.8–1.4)	**1.4 (1.0–1.9)**	1.6 (1.1–2.2) *	1.1 (0.7–1.6)	0.9 (0.5–1.7)	0.9 (0.6–1.5)	1.3 (0.7–2.3)	1.9 (1.8–4.7) *	1.3 (0.8–1.9)	1.0 (0.6–1.6)
**Histopathological Subtype**												
SSM ^1^	1.0 (ref)	1.0 (ref)	1.0 (ref)	1.0 (ref)	1.0 (ref)	1.0 (ref)	1.0 (ref)	1.0 (ref)	1.0 (ref)	1.0 (ref)	1.0 (ref)	1.0 (ref)
NM ^2^	0.8 (0.7–1.1)	0.8 (0.6–1.1)	0.9 (0.7–1.2)	0.8 (0.6–1.1)	0.9 (0.7–1.2)	0.9 (0.6–1.3)	0.8 (0.5–1.3)	1.2 (0.8–1.8)	0.9 (0.5–1.5)	0.8 (0.5–1.3)	0.9 (0.6–1.4)	1.0 (0.6–1.5)
ALM ^3^	0.9 (0.5–1.9)	0.5 (0.2–1.4)	0.6 (0.3–1.5)	0.5 (0.2–1.3)	0.5 (0.2–1.2)	1.2 (0.5–3.2)	0.0 (0.0–0.0)	0.9 (0.3–3.3)	1.3 (0.4–4.7)	0.2 (0.0–1.7)	0.5 (0.2–2.4)	1.0 (0.3–3.6)
Other	0.9 (0.5–1.5)	1.1 (0.6–1.8)	0.8 (0.4–1.4)	0.8 (0.4–1.4)	0.8 (0.5–1.5)	1.4 (0.7–2.7)	0.7 (0.2–2.0)	1.1 (0.5–2.6)	0.6 (0.2–1.7)	0.6 (0.2–2.0)	0.8 (0.3–2.2)	0.7 (0.3–2.1)
MM ^4^ unspecified	1.0 (0.7–1.4)	1.0 (0.8–1.5)	0.9 (0.6–1.2)	0.9 (0.7–1.3)	0.9 (0.6–1.2)	1.0 (0.6–1.6)	0.8 (0.4–1.6)	1.0 (0.6–1.7)	1.2 (0.7–2.1)	1.1 (0.6–1.9)	1.3 (0.8–2.1)	1.0 (0.6–1.8)
**Breslow Thickness**	1.0 (1.0–1.0)	1.0 (1.0–1.0)	1.0 (0.9–1.0)	1.0 (1.0–1.0)	1.0 (0.9–1.0)	1.0 (0.9–1.0)	1.0 (1.0–1.1)	1.0 (0.9–1.0)	**0.9 (0.8–1.0)**	1.0 (1.0–1.1)	1.0 (1.0–1.1)	1.0 (0.9–1.0)
**Ulceration Status**												
Ulcerated	1.0 (0.8–1.4)	1.0 (0.7–1.3)	1.3 (1.0–1.6)	1.1 (0.9–1.5)	1.1 (0.8–1.5)	1.0 (0.7–1.4)	0.9 (0.6–1.5)	0.9 (0.6–1.3)	1.0 (0.7–1.7)	0.8 (0.5–1.2)	0.7 (0.5–1.1)	1.4 (0.9–2.0)
Not ulcerated	1.0 (ref)	1.0 (ref)	1.0 (ref)	1.0 (ref)	1.0 (ref)	1.0 (ref)	1.0 (ref)	1.0 (ref)	1.0 (ref)	1.0 (ref)	1.0 (ref)	1.0 (ref)

^1^, superficial spreading melanoma; ^2^, nodular melanoma; ^3^, acral lentiginous melanoma; and ^4^, malignant melanoma. Numbers in bold represent *p*-values < 0.05. * indicates statistical significance after correcting for multiple comparisons using a false discovery rate of 0.05.

## Data Availability

The data presented in this study are available on request from the corresponding authors. The data are provided by the Netherlands Cancer Registry to the Erasmus MC Cancer Institute and are therefore not publicly available.

## Supplementary Materials

The following are available online at https://www.mdpi.com/article/10.3390/cancers13174431/s1, Table S1: Categorization of the topography codes of distant metastases according to the ICD-O for analysis, Table S2: Sentinel lymph node procedure results before and after the implementation of adjuvant systemic therapy in patients with regional lymph node metastases from 01/01/2014, Table S3: The mean time interval from diagnosis of primary melanoma to stage IV progression in relation to patient and tumour characteristics of patients diagnosed with a primary melanoma from July 2017 onwards in a multivariate model, Table S4: Patient and tumour characteristics of the primary melanoma of stage IV melanomas stratified for each site of distant metastasis and Table S5: The localisation of distant metastases in relation to patient and tumour characteristics in a univariate model with the lungs as the reference metastasized site.
