# Supplementary material for: Primary Melanoma Characteristics of Metastatic Disease: A Nationwide Cancer Registry Study
Source: Cancers (Basel). 2021 Sep 2;13(17):4431. doi: 10.3390/cancers13174431 (PMC8431672; doi:10.3390/cancers13174431)
Supplement: Supplementary file 1 [file cancers-13-04431-s001.zip › cancers-1350871-supplementary.pdf]

cancers MDPI

Supplementary Materials

Primary Melanoma Characteristics of Metastatic Disease: A Nationwide Cancer Registry Study

Catherine Zhou [1], Marieke Louwman [2], Marlies Wakkee [1], Astrid van der Veldt [3], Dirk Grünhagen [4], Cornelis Verhoef [4], Antien Mooyaart [5], Tamar Nijsten [1] and Loes Hollestein [1,2,*]

[1] Department of Dermatology, Erasmus MC Cancer Institute, Doctor Molewaterplein 40, 3015 GD Rotterdam, The Netherlands; c.zhou@erasmusmc.nl (C.Z.); m.wakkee@erasmusmc.nl (M.W.); t.nijsten@erasmusmc.nl (T.N.); l.hollestein@erasmusmc.nl (L.H.)

[2] Department of Research and Development, Netherlands Comprehensive Cancer Organization, Godebaldkwartier 419, 3511 DT Utrecht, The Netherlands; m.louwman@iknl.nl (M.L.); l.hollestein@erasmusmc.nl (L.H.)

[3] Department of Medical Oncology and Radiology & Nuclear Medicine, Erasmus MC Cancer Institute, Doctor Molewaterplein 40, 3015 GD Rotterdam, The Netherlands; a.vanderveldt@erasmusmc.nl

[4] Department of Surgical Oncology, Erasmus MC Cancer Institute, Doctor Molewaterplein 40, 3015 GD Rotterdam, The Netherlands; d.grunhagen@erasmusmc.nl (D.G.); c.verhoef@erasmusmc.nl (C.V.)

[5] Department of Pathology, Erasmus MC Cancer Institute, Doctor Molewaterplein 40, 3015 GD Rotterdam, The Netherlands; a.mooyaart@erasmusmc.nl

* Correspondence: l.hollestein@erasmusmc.com; Tel.: +31 6 50 03 24 07

Table S1. Categorization of the topography codes of distant metastases according to the International Classification of Diseases for Oncology (ICD-O) for analysis.

ICD-O code	Organ tract
C16<br>C17<br>C18<br>C20<br>C23<br>C25	Gastrointestinal tract
C22	Liver
C34	Lung
C38	Heart and pleura
C40<br>C41	Bone
C42	Splene
C44	Skin
C48	Peritoneum
C49	Connective tissue
C71	Brain
C74	Adrenal gland
C77	Lymph node
C01<br>C07<br>C09<br>C10<br>C13<br>C14<br>C15	Other

cancers MDPI

C24
C26
C32
C33
C47
C50
C52
C53
C54
C56
C61
C64
C65
C67
C69
C70
C72
C73
C75
C76
C80

cancers

MDPI

Table S2. Sentinel lymph node biopsy results before and after the implementation of adjuvant systemic therapy in patients with regional lymph node metastases from January 2014 (continues next page).

	Before the implementation of adjuvant systemic therapy				
	**Total, No.**	**SLNB performed, No. (% of total)**	**SLNB-positive, No. (% of SLNB performed)**	**Isolated tumour cells, No. (% of SLNB performed)**	**Micrometastasis, No. (% of SLNB performed)**
All patients regardless of eligibility for an SLNB	1462	754 (51.6)	231 (30.6)	30 (4.0)	109 (7.5)
No clinical suspicion for metastases	1258	719 (57.2)	209 (29.1)	30 (4.2)	104 (14.4)
No clinical suspicion for metastases and ≥ pT1b	1156	705 (61.0)	204 (28.9)	30 (4.3)	103 (14.6)
**Pathological T-stage of patients without clinical suspicion for metastases**					
pT1a	70	3 (4.3)	0 (0.0)	0 (0.0)	1 (33.3)
pT1b	69	31 (44.9)	2 (6.5)	2 (6.5)	3 (9.7)
pT2a	222	131 (59.0)	25 (19.1)	11 (8.4)	13 (9.9)
pT2b	69	46 (66.7)	10 (21.7)	2 (4.3)	5 (10.9)
pT3a	213	142 (66.7)	46 (32.4)	5 (3.5)	20 (14.1)
pT3b	205	145 (70.7)	49 (33.8)	8 (5.5)	27 (18.6)
pT4a	144	82 (56.9)	31 (37.8)	0 (0.0)	7 (8.5)
pT4b	234	128 (54.7)	43 (33.6)	2 (1.6)	28 (21.9)
Unknown	32	11 (34.4)	3 (27.3)	0 (0.0)	0 (0.0)

cancers MDPI

	After the implementation of adjuvant systemic therapy				
	Total, No.	SLNB performed, No. (% of total)	SLNB-positive, No. (% of SLNB performed)	Isolated tumour cells, No. (% of SLNB performed)	Micrometastasis, No. (% of SLNB performed)
All patients regardless of eligibility for an SLNB	668	464 (69.5)	185 (39.9)	55 (11.9)	175 (37.7)
No clinical suspicion for metastases	518	442 (85.3)	168 (38.0)	54 (12.2)	172 (38.9)
No clinical suspicion for metastases and ≥ pT1b	498	434 (87.1)	163 (37.6)	54 (12.4)	169 (38.9)
**Pathological T-stage of patients without clinical suspicion for metastases**					
pT1a	7	2 (28.6)	2 (100)	0 (0.0)	0 (0.0)
pT1b	33	31 (93.3)	7 (22.6)	6 (19.4)	16 (51.6)
pT2a	122	114 (93.4)	28 (24.6)	16 (14.0)	65 (57.0)
pT2b	21	20 (95.2)	6 (30.0)	2 (10)	11 (55)
pT3a	96	89 (92.7)	32 (36.0)	12 (13.5)	30 (33.7)
pT3b	80	70 (87.5)	35 (50.0)	7 (10)	21 (30)
pT4a	47	40 (85.1)	19 (47.5)	5 (12.5)	6 (15)
pT4b	99	70 (70.7)	36 (51.4)	6 (8.6)	20 (28.6)
Unknown	13	6 (46.2)	3 (50.0)	0 (0.0)	3 (50.0)

cancers MDPI

Table S3. The mean time interval from diagnosis of primary melanoma to first stage IV presentation in relationship to patient and tumour characteristics of patients diagnosed with a single stage I-III primary melanoma in July 2017-December 2019 in a multivariate model.

	No.	Mean time interval, months (SD[1])	Difference in months	95% CI[2]	*p*-value
No. of stage I-III patients with progression to stage IV	254				
**Gender**					
Male	164	10.2 (5.8)	*ref*		
Female	90	10.8 (6.5)	0.6	-0.94 to 2.19	0.43
**Age at diagnosis**					
< 40	10	11.0 (5.9)	1.0	-2.95 to 4.97	0.62
41-50	25	10.7 (6.2)	0.7	-1.93 to 3.39	0.59
51-60	32	11.1 (6.7)	1.1	-1.34 to 3.51	0.90
61-70	75	10.5 (6.6)	0.5	-1.33 to 2.28	0.54
> 71	112	10.0 (5.6)	*ref*		
**Site primary melanoma**					
Face	27	10.4 (5.5)	0.0	-2.56 to 2.25	1.00
Scalp and neck	32	8.5 (6.7)	-1.8	-4.23 to 0.54	0.13
Trunk	113	10.4 (6.1)	*ref*		
Upper extremities and shoulders	29	10.6 (5.7)	0.2	-2.24 to 2.72	0.85
Lower extremities and hips	52	11.4 (6.0)	1.3	-0.95 to 3.04	0.30
**Histopathological subtype**					
Superficial spreading	137	10.1 (6.1)	*ref*		
Nodular melanoma	73	10.1 (6.1)	-0.5	-2.26 to 1.17	0.53
Acral lentiginous melanoma	4	9.5 (8.8)	-1.1	-7.21 to 4.94	0.71
Other	14	11.9 (6.4)	1.3	-2.07 to 4.65	0.45
Malignant melanoma unspecified	23	9.1 (6.0)	-1.5	-4.25 to 1.15	0.26
**Breslow thickness**					
< 1.00 mm	19	9.2 (6.07)	-0.5	-3.44 to 2.37	0.72
1.01-2.00 mm	48	13.2 (6.4)	3.4	1.41 to 4.44	<0.001
2.01-4.00 mm	73	9.9 (5.4)	0.1	-1.64 to 1.87	0.90
> 4.01 mm	100	9.8 (6.0)	*ref*		

[1], standard deviation; [2], confidence interval.

cancers

Table S4. Patient- and tumour characteristics of the primary melanoma of stage IV melanomas stratified for each site of distant metastasis.

	Lungs	Lymph node	Liver	Brain	Bone	Connective tissue	Other	Spleen	Adrenal gland	Heart or pleura	Skin	Peritoneum	Digestive system	Total
**Gender, No. (%)**														
Male	587 (64)	452 (65)	350 (66)	398 (63)	320 (66)	239 (56)	143 (60)	86 (74)	119 (68)	71 (58)	73 (60)	100 (61)	100 (65)	3038 (64)
Female	327 (36)	242 (35)	179 (34)	230 (37)	168 (34)	189 (44)	96 (40)	31 (27)	55 (32)	52 (42)	48 (40)	64 (39)	55 (35)	1736 (36)
**Age at diagnosis, mean (SD [1]), y**	61 (14)	61 (14)	61 (14)	59 (14)	60 (14)	59 (13)	58 (14)	59 (14)	62 (13)	60 (14)	58 (15)	58 (14)	60 (13)	60 (14)
**Site primary melanoma, No. (%)**														
Head/neck	160 (18)	86 (12)	98 (19)	105 (17)	80 (17)	58 (14)	37 (16)	21 (18)	24 (14)	33 (27)	13 (11)	17 (10)	17 (11)	749 (16)
Trunk	453 (50)	310 (45)	256 (49)	319 (51)	225 (46)	188 (44)	118 (50)	61 (52)	89 (51)	49 (40)	46 (38)	86 (52)	89 (57)	2289 (48)
Upper extremities	145 (16)	103 (15)	78 (15)	99 (16)	82 (17)	76 (18)	36 (15)	20 (17)	35 (20)	18 (15)	21 (18)	27 (17)	20 (13)	760 (16)
Lower extremities	153 (17)	193 (28)	95 (18)	103 (17)	99 (20)	104 (24)	45 (19)	15 (13)	26 (15)	23 (19)	40 (33)	34 (21)	29 (19)	959 (20)
Missing	3 (0)	2 (0)	3 (1)	2 (0)	2 (0)	2 (0)	0 (0)	0 (0)	0 (0)	0 (0)	1 (0)	0 (0)	0 (0)	17 (0)
**Histopathological subtype, No. (%)**														
SSM [2]	497 (54)	395 (57)	295 (56)	368 (59)	284 (58)	253 (59)	135 (57)	71 (61)	95 (55)	72 (59)	70 (58)	94 (57)	91 (59)	2720 (57)
NM [3]	247 (27)	164 (24)	129 (24)	158 (25)	119 (24)	104 (24)	55 (23)	29 (25)	49 (28)	25 (20)	28 (23)	37 (23)	37 (24)	1181 (25)
ALM [4]	18 (2)	19 (3)	6 (1)	8 (1)	6 (1)	6 (1)	6 (3)	0 (0)	3 (2)	3 (2)	1 (0)	2 (1)	3 (2)	81 (2)
Other	41 (5)	26 (4)	27 (5)	21 (3)	17 (4)	14 (3)	13 (5)	4 (3)	8 (5)	4 (3)	3 (3)	5 (3)	4 (3)	187 (4)
MM[5] unspecified	111 (12)	90 (13)	72 (14)	73 (12)	62 (13)	51 (12)	30 (13)	13 (11)	19 (11)	19 (15)	19 (16)	26 (16)	20 (13)	605 (13)
Missing	0 (0)	0 (0)	0 (0)	0 (0)	0 (0)	0 (0)	0 (0)	0 (0)	0 (0)	0 (0)	0 (0)	0 (0)	0 (0)	0 (0)
**Breslow thickness, mean (SD), mm**	3.5 (3.2)	3.4 (3.0)	3.6 (3.4)	3.2 (3.0)	3.4 (3.1)	3.2 (3.2)	3.2 (3.2)	3.5 (3.1)	3.1 (3.1)	2.5 (2.4)	3.5 (3.2)	3.1 (3.3)	2.9 (2.7)	3.3 (3.1)
Missing, No. (%)	63 (7)	56 (8)	39 (7)	47 (8)	44 (9)	32 (7)	19 (9)	12 (11)	11 (6)	8 (7)	12 (10)	15 (9)	10 (6)	368 (8)

cancers MDPI

**Ulceration status, No. (%)**														
Ulcerated	307 (34)	232 (33)	170 (32)	222 (35)	167 (34)	143 (33)	72 (30)	34 (29)	48 (28)	34 (28)	34 (28)	41 (25)	56 (36)	1560 (33)
Not ulcerated	482 (53)	353 (51)	274 (52)	312 (50)	243 (50)	217 (51)	129 (54)	60 (51)	95 (55)	69 (56)	64 (53)	96 (59)	79 (51)	2473 (52)
Missing	125 (14)	109 (16)	85 (16)	94 (15)	78 (16)	68 (16)	38 (16)	23 (20)	31 (18)	20 (16)	23 (19)	27 (16)	20 (13)	741 (16)
**Total, No.**	914	694	529	628	488	428	239	117	174	123	121	164	155	4774

[1], standard deviation; [2], superficial spreading melanoma; [3], nodular melanoma; [4], acral lentiginous melanoma; and [5], malignant melanoma.

cancers

MDPI

Table S5. The localization of distant metastases in relationship to patient- and tumour characteristics in a univariate model with the lungsas the reference metastatic site.

	Lymph node	Liver	Brain	Bone	Connec-tive tissue	Other	Spleen	Adrenal gland	Heart/ mediasti- num	Skin	Perito-neum	Digestive system
	OR (95% CI)	OR (95% CI)	OR (95% CI)	OR (95% CI)	OR (95% CI)	OR (95% CI)	OR (95% CI)	OR (95% CI)	OR (95% CI)	OR (95% CI)	OR (95% CI)	OR (95% CI)
**Gender**												
Male	1.0 (ref)	1.0 (ref)	1.0 (ref)	1.0 (ref)	1.0 (ref)	1.0 (ref)	1.0 (ref)	1.0 (ref)	1.0 (ref)	1.0 (ref)	1.0 (ref)	1.0 (ref)
Female	1.0 (0.8-1.2)	0.9 (0.7-1.2)	1.0 (0.8-1.3)	0.9 (0.7-1.2)	**1.4 (1.1-1.8)**	1.2 (0.9-1.6)	**0.6 (0.4-1.0)**	0.8 (0.6-1.2)	1.3 (0.9-1.9)	1.2 (0.8-1.7)	1.1 (0.8-1.6)	1.0 (0.7-1.4)
**Age at diagnosis**	1.0 (1.0-1.0)	1.0 (1.0-1.0)	**1.0 (1.0-1.0)**	**1.0 (1.0-1.0)**	**1.0 (1.0-1.0)**	**1.0 (1.0-1.0)**	1.0 (1.0-1.0)	1.0 (1.0-1.0)	1.0 (1.0-1.0)	1.0 (1.0-1.0)	1.0 (1.0-1.0)	1.0 (1.0-1.0)
**Body site**												
Head/neck	0.8 (0.6-1.1)	1.1 (0.8-1.5)	0.9 (0.7-1.2)	1.0 (0.7-1.4)	0.9 (0.6-1.2)	0.9 (0.6-1.4)	1.0 (0.6-1.7)	0.8 (0.5-1.2)	**1.9 (1.2-3.1)**	0.8 (0.4-1.5)	**0.6 (0.3-1.0)**	**0.5 (0.3-0.9)**
Trunk	1.0 (ref)	1.0 (ref)	1.0 (ref)	1.0 (ref)	1.0 (ref)	1.0 (ref)	1.0 (ref)	1.0 (ref)	1.0 (ref)	1.0 (ref)	1.0 (ref)	1.0 (ref)
Upper extremities	1.0 (0.8-1.4)	0.9 (0.7-1.3)	1.0 (0.7-1.3)	1.1 (0.8-1.6)	1.3 (0.9-1.8)	0.9 (0.6-1.4)	1.0 (0.6-1.8)	1.3 (0.8-0.9)	1.1 (0.7-2.0)	1.4 (0.8-2.5)	1.0 (0.6-1.6)	0.7 (0.4-1.2)
Lower extremities	**1.8 (1.4-2.4)**	1.1 (0.8-1.5)	1.0 (0.7-1.3)	**1.3 (1.0-1.8)**	**1.6 (1.2-2.2)**	1.1 (0.8-1.7)	0.7 (0.4-1.3)	0.9 (0.5-1.4)	1.4 (0.8-2.3)	**2.6 (1.6-4.0)**	1.2 (0.8-1.8)	1.0 (0.6-1.5)
**Histopathological subtype**												
SSM [1]	1.0 (ref)	1.0 (ref)	1.0 (ref)	1.0 (ref)	1.0 (ref)	1.0 (ref)	1.0 (ref)	1.0 (ref)	1.0 (ref)	1.0 (ref)	1.0 (ref)	1.0 (ref)
NM [2]	0.8 (0.7-1.1)	0.9 (0.7-1.1)	0.9 (0.7-1.1)	0.8 (0.6-1.1)	0.8 (0.6-1.1)	0.8 (0.6-1.2)	0.8 (0.5-1.3)	1.0 (0.7-1.5)	0.7 (0.4-1.1)	0.8 (0.5-1.3)	0.8 (0.5-1.2)	0.8 (0.5-1.2)

ALM [3]	1.3 (0.7-2.6)	0.6 (0.2-1.4)	0.6 (0.3-1.4)	0.6 (0.2-1.5)	0.7 (0.3-1.7)	1.2 (0.5-3.2)	n/a	0.9 (0.3-3.0)	1.2 (0.3-4.0)	0.4 (0.1-3.0)	0.6 (0.2-2.6)	0.9 (0.3-3.2)
Other	0.8 (0.5-1.3)	1.1 (0.7-1.8)	0.7 (0.4-1.2)	0.7 (0.4-1.3)	0.7 (0.4-1.3)	1.2 (0.6-2.2)	0.7 (0.3-2.0)	0.7 (0.4-1.3)	0.7 (0.2-2.0)	0.5 (0.2-1.7)	0.6 (0.2-1.7)	0.5 (0.2-1.5)
MM [4] unspecified	1.0 (0.8-1.4)	1.1 (0.8-1.5)	0.9 (0.6-1.2)	1.0 (0.7-1.4)	0.9 (0.6-1.3)	1.0 (0.6-1.5)	0.8 (0.4-1.5)	0.9 (0.5-1.5)	1.2 (0.7-2.0)	1.2 (0.7-2.1)	1.2 (0.8-2.0)	1.0 (0.6-1.7)
Breslow thickness	1.0 (1.0-1.0)	1.0 (1.0-1.0)	1.0 (0.9-1.0)	1.0 (1.0-1.0)	1.0 (0.9-1.0)	1.0 (0.9-1.0)	1.0 (1.0-1.1)	1.0 (0.9-1.0)	**0.9 (0.8-1.0)**	1.0 (1.0-1.1)	1.0 (1.0-1.1)	1.0 (0.9-1.0)
**Ulceration status**												
Ulcerated	1.0 (0.8-1.3)	1.0 (0.8-1.3)	1.1 (0.9-1.4)	1.1 (0.9-1.4)	1.0 (0.8-1.3)	0.9 (0.7-1.2)	0.9 (0.6-1.5)	0.8 (0.6-1.2)	0.8 (0.5-1.1)	0.8 (0.5-1.3)	0.7 (0.5-1.1)	1.2 (0.8-1.7)
Not ulcerated	1.0 (ref)	1.0 (ref)	1.0 (ref)	1.0 (ref)	1.0 (ref)	1.0 (ref)	1.0 (ref)	1.0 (ref)	1.0 (ref)	1.0 (ref)	1.0 (ref)	1.0 (ref)

[1], superficial spreading melanoma; [2], nodular melanoma; [3], acral lentiginous melanoma; and [4], malignant melanoma. Numbers in bold represent p-values < 0.
